# Indices of Abdominal Adiposity and Cardiorespiratory Fitness Test Performance in Middle-School Students

**DOI:** 10.1155/2013/912460

**Published:** 2013-03-04

**Authors:** Ryan Burns, James C. Hannon, Timothy A. Brusseau, Barry Shultz, Patricia Eisenman

**Affiliations:** Department of Exercise and Sport Science, University of Utah, College of Health, 250 S. 1850 E., HPER North RM 241, Salt Lake City, UT 84112, USA

## Abstract

*Background*. Previous research suggests that use of BMI as a screening tool to assess health in youth has limitations. Valid alternative measures to assess body composition are needed to accurately identify children who are aerobically fit, which is an indicator of health status. The purpose of this study was to examine the associations between select anthropometric measures and cardiorespiratory fitness test performance in middle-school students. *Methods*. Participants included 134 students (65 boys and 69 girls) recruited from the 6th, 7th, and 8th grades. Anthropometric measures consisted of BMI, waist circumference (WC), waist-to-height ratio (WHtR), and percent body fat estimated from two-site skinfolds (%BF-SKF), as well as the hand-held OMRON BIA device (%BF-BIA). Cardiorespiratory fitness tests included the one-mile run and PACER test. Data were collected on four separate testing days during the students' physical education classes. *Results.* There were statistically significant moderate correlations between the %BF estimations, WHtR, and cardiorespiratory fitness test scores in both genders (*P* < .001). BMI at best only displayed weak correlations with the cardiorespiratory fitness test scores. *Conclusions*. The results suggest that alternative measures such as %BF-SKF, %BF-BIA, and WHtR may be more valid indicators of youth aerobic fitness lending to their preferred use over BMI.

## 1. Introduction

The current pediatric obesity epidemic manifests concerns for adverse cardiovascular risk factors among overweight youth. However, Eisenmann et al. [1], using body mass index (BMI) as the marker of adiposity, found that youth in both the low- and high-BMI categories were associated with a more favorable cardiovascular disease (CVD) risk-factor profile than individuals whose BMIs were in the “healthy” range. This paradox leads to a significant issue in assessing health and fitness in youth when using BMI. Research has also suggested that along with body composition, aerobic fitness must also be considered to accurately assess health status in a population. Lee et al. [2] found that unfit lean men had a higher risk of cardiovascular disease and all-cause mortality than fit but overweight men. These findings suggest that fitness offers some protection against CVD risk even if the individual is overweight. Similar results have been reported for the female population [3]. Using skinfold thickness as the measure of body fatness and stratifying youth into high-fat/high-fitness, high-fat/low-fitness, low-fat/high-fitness, and low-fat/low-fitness groups, it was found that both fitness and fatness must be considered to assess CVD risk in the pediatric population [4]. Jago et al. [5] found that fitness and fatness influenced CVD risk, but body fatness was the stronger predictor of health risk in a sample consisting of 6th grade youth. Acknowledging that both body composition and cardiorespiratory fitness are both important in determining the health status of an individual, and that BMI may have inherent limitations that affect its validity as a marker of adiposity, a question must be addressed to determine what alternative body composition measures to BMI most strongly associate with cardiorespiratory fitness in the pediatric population.

 Although it is used extensively in epidemiological research, BMI has its limitations. The most prominent is that it does not take into account lean body mass nor does it specify the degree of central adiposity [6], which has been linked to increased risks of chronic disease in boys and girls [7, 8]. Although BMI is commonly used and easy to calculate, it is uncertain whether it is superior to other feasible measures of childhood body composition [9]. 

 One alternative to BMI that has demonstrated utility in estimating body composition and chronic disease risk in youth is skinfold thickness assessment (SKF). The two-site SKF (tricep, calf) has yielded body fat estimates that have agreed closely with a four-component criterion measure of body fat in an independent sample of Caucasian and African American adolescents [10]. However, if used as the primary assessment in physical education or even in clinical settings, a child being tested may become uncomfortable due to the intrusive nature of the assessment (skin pinching). SKF is also time consuming; so, assessing a large number of children within a restricted time frame may be cumbersome for both physical educators and researchers alike. 

 Other feasible alternatives for body composition assessment include bioelectrical impedance analysis (BIA), waist circumference, and waist-to-height ratio. BIA has shown acceptable and reliable results when predicting percent body fat [11, 12]. Ihmels et al. [13] found that the Omron handheld BIA device and the two-site SKF for assessing body composition produced agreeable results with each other. Another alternative body composition measure is waist circumference (WC). Bassali et al. [14] had shown that children with a WC above the 90th percentile are at higher risk for dyslipidemia and insulin resistance compared to obese children, as determined by BMI, with a normal WC. Another measure of central adiposity, waist-to-height ratio (WHtR), has the advantage of not requiring population-specific, age, and sex-specific reference tables contrary to WC [15]. In Mexican children aged 6–12 years, a WHtR cutoff of .59 demonstrated to be a strong predictor of metabolic syndrome, with values below .5 displaying poor sensitivity and specificity [16]. WHtR has also been shown to detect adverse CVD risk factors in normal weight children who are centrally obese [17]. In the US adult population, area under the curve (AUC) values, which are used to identify the accuracy of a test or measure's ability to classify individuals with or without a disease or condition, were higher for WHtR than all other anthropometric parameters in detecting cardiometabolic conditions in both women and men [18].

 All of the aforementioned anthropometric measures have displayed varying degrees of association with health markers in the pediatric and adult populations. To our knowledge, no previous research has compared all of these measures' relationships to youth cardiorespiratory fitness, which has been linked to health status in both children and adults [19, 20]. Two primary components of physical fitness include cardiorespiratory fitness and muscular fitness; however, it is cardiorespiratory fitness that is more closely linked to health, specifically cardiometabolic health. Cardiorespiratory fitness, also called cardiovascular fitness, aerobic fitness, or aerobic capacity, is the overall capacity of the cardiovascular and respiratory systems to carry out prolonged exercise. 

 Two popular aerobic capacity fitness tests used in physical education settings include the one-mile run and the 20 m Progressive Aerobic Cardiovascular Endurance Run (PACER). Both of these field tests estimate aerobic capacity, which can be operationally defined as estimated maximal oxygen uptake or VO_2_ max. To interpret the scores, the PACER test is converted to one-mile run times via the Primary Field Test Equating Method [21], which is then converted to estimated VO_2_ max via the Cureton et al. [22] equation. Actual one-mile run times, a more direct assessment of aerobic capacity, are also converted to VO_2_ max using this same equation. Welk et al. [23] demonstrated that aerobic fitness (VO_2_ max) could be used to differentiate between American adolescents with and without metabolic syndrome. Mesa et al. [24] showed that higher levels of cardiorespiratory fitness are associated with a more favorable metabolic profile in both overweight and nonoverweight Spanish adolescents. Cardiorespiratory fitness has also been inversely associated with low-grade inflammatory markers [25]. 

 Due to the associations between cardiorespiratory fitness and health, it is necessary to find relationships between anthropometric measures that estimate body composition with aerobic fitness test performance to more clearly understand what screening measures have utility identifying youth with inadequate cardiorespiratory fitness and consequent increased risk for chronic disease. Anthropometric measures that correlate most strongly to cardiorespiratory fitness can serve as proxy measures of health status for physical education specialists, school nurses, and clinicians to use in a variety of settings. Therefore, the primary purpose of this investigation was to examine the relationships between body composition estimated from select anthropometric measures and cardiorespiratory fitness in middle school students. The specific anthropometric measurements examined consisted of BMI, WC, WHtR, and percent body fat estimated from two-site skinfolds (%BF-SKF) and the Omron hand-BIA device (%BF-BIA). The one-mile run and PACER tests were used to assess cardiorespiratory fitness. It was hypothesized that measures of abdominal adiposity (WC and WHtR) and body fat percentage estimations (%BF-SKF and %BF-BIA) would have higher associations with cardiorespiratory fitness than BMI based on the previous research examining these parameters' relationships with health status in youth. 

## 2. Methods

### 2.1. Participants

 Participants included 134 school-aged youth (65 boys and 69 girls) recruited from the 6th, 7th, and 8th grades (mean age = 12.9 years, SD = .07 years) from three schools located in a metropolitan area in the Southwestern United States. The sample distribution by grade included 34 6th graders (17 boys and 17 girls), 52 7th graders (22 boys and 30 girls), and 48 8th graders (26 boys and 22 girls). Written consent was obtained from parents and assent was obtained from the participants prior to data collection. The University IRB and principals from the participating schools approved the protocols used in this study.

### 2.2. Procedures

 All data collection took place during each student's physical education class on 4 separate testing days with at least 1 week separating testing sessions. All anthropometric and cardiorespiratory fitness assessments were conducted at least 2 hours postprandial during the final two class periods of the school day. A trained graduate student within the Department of Exercise and Sport Science administered all anthropometric measures and fitness tests to ensure consistency during data collection. Body composition and anthropometric measures were administered on Day 1. Students were asked to remove their shoes, as height (to the nearest 1 cm) and weight (to the nearest 0.1 kg) were determined using a portable stadiometer (Seca 213; Chino, CA, USA) and medical scale (Tanita HD-314; Arlington Heights, IL, USA). Students then entered a private screening area where three abdominal circumference measurements were taken at the level of the superior border of the iliac crest on the participant's right side using a steel measuring tape. All measurements were estimated to the nearest 0.1 cm with the average of the three measures used as the participant's waist circumference. Skinfold measurements were taken on the students' tricep and calf using a Lange (Ann Arbor, MI, USA) skinfold caliper. Each site was measured 3 times in a rotating order on the students' right side with the average used as the recorded measure. Percent body fat was then estimated using the equations from Slaughter et al. [26]. Finally, the students' height, weight, age, and gender were entered into a handheld OMRON body fat analyzer (Model HBF-306; Lake Forest, IL, USA). The students then held the analyzer with arms extended, parallel to the floor until the device displayed the student's body fat percentage.

 The 20 m PACER test was administered on Day 2. The PACER test was administered on a marked gymnasium floor with background music and cadence given by an audio CD. No more than 10 students participated in the assessment at any given time. Students' ran from one floor marker to another marker set 20 m apart while keeping pace with the prerecorded cadence. The test was terminated when a student twice failed to reach the opposite marker in the allotted time frame or when he/she voluntarily stopped. Day 3 consisted of the one-mile run test. The one-mile run was administered on either a standard track or measured flat trail on school grounds. No more than 10 students participated in the one-mile run at any time. Time was kept via a handheld stopwatch (Robic Oslo M427; Oxford, CT, USA). Finally, Day 4 served as the makeup day for those students who had not completed a test in Day 1 through Day 3.

### 2.3. Statistical Analysis

 Data were screened for outliers and normality was checked prior to the main analyses. Comparisons among grade levels and between the genders on anthropometric measures and cardiorespiratory fitness test performance were examined using multiple 2 × 3 factorial ANOVA tests followed by Bonferroni post hoc analyses. Canonical correlations were used to examine linear weighted associations between the anthropometric multivariable dimension (BMI, WC, WHtR, %BF-SKF, and %BF-BIA) and the cardiorespiratory fitness multivariable dimension (PACER and one-mile run). Significantly correlated dimensions (or variates) were reported along with the Redundancy Index (Rd) for each significant canonical function, which was used as an estimate of the amount of shared variance between anthropometric dimension (independent variate) and the cardiorespiratory fitness dimension (dependent variate). Based on the practical significance of the Rd, the standardized coefficients, canonical loadings (the correlation between a measure and its variate), and canonical crossloadings (the correlation between a measure and the opposite variate) were reported. Pearson product-moment correlations were then employed to examine the associations among the specific anthropometric measures and between the anthropometric measures and cardiorespiratory fitness test scores within each gender group. BMI and age were then controlled for using partial correlations. Statistical significance was set at an alpha level of *P* ≤ .05 and adjusted appropriately for ANOVA post hoc analysis. Data analyses were carried out using STATA v12.0 (College Station, TX, USA) statistical software.

## 3. Results

### 3.1. Grade and Gender Differences

Table 1 shows the means, standard deviations, grade, and gender effects for the anthropometric measures and cardiorespiratory fitness test scores per grade and gender group. A factorial ANOVA test revealed a significant grade effect for height (*F*_(2,128)_ = 29.85, *P* < .001) and weight (*F*_(2,128)_ = 27.52, *P* < .001). Students in grade 7 were significantly taller (*M* = 1.63 m, SD = .10 m) and heavier (*M* = 49.7 kg, SD = 10.0 kg) than students in grade 6 (*M* = 1.52 m, SD = .06 m, height; *M* = 40.6 kg, SD = 7.26 kg, weight) (*P* < .01), and students in grade 8 were significantly taller (*M* = 1.66 m, SD = .08 m) than students in grade 6 (*P* < .01), and significantly heavier (*M* = 57.4 kg, SD = 12.0 kg) than students in grade 7 and grade 6 (*P* < .01). There was also a significant grade effect for BMI (*F*_(2,128)_ = 12.04, *P* < .001) as BMI was higher in grade 8 (*M* = 20.4 kg/m^2^, SD = 2.90 kg/m^2^) compared to grade 7 (*M* = 18.4 kg/m^2^, SD = 2.59 kg/m^2^) and grade 6 (*M* = 17.4 kg/m^2^, SD = 2.95 kg/m^2^) (*P* < .001). A grade effect for WC (*F*_(2,128)_ = 6.67, *P* < .001) revealed that measurements were higher in grade 8 (*M* = 70.0 cm, SD = 8.27 cm) than grade 6 (*M* = 63.5 cm, SD = 6.13 cm) (*P* < .001) but not grade 7 (*M* = 66.8 cm, SD = 7.77 cm). There were no statistically significant differences among the grades in WHtR, %BF-SKF, or %BF-BIA. Regarding the cardiorespiratory fitness test scores, a grade effect for one-mile run times (*F*_(2,128)_ = 9.85, *P* < .001) revealed that grade 8 had significantly faster one-mile run times (*M* = 447.7 s, SD = 82.94 s) than grade 7 (*M* = 451.2 s, SD = 71.3 s) and grade 6 (*M* = 485.5 s, SD = 136.2 s) (*P* < .01); however, there were no differences among grades in PACER test performance. 

 Regarding the gender effects, boys in the sample were taller and heavier than girls (*P* < .05); however, there were no differences between the genders in BMI. Boys also displayed lower body fat percentages than girls when estimated from %BF-SKF and %BF-BIA (*P* < .001), but there were no statistically significant differences between the genders in WC or WHtR. Finally, regarding the cardiorespiratory fitness test scores, boys had statistically faster one-mile run times (*P* < .001) and higher PACER scores than girls (*P* < .001). There was no statistically significant grade by gender interactions.

### 3.2. Canonical Correlations

Table 2 depicts the two statistically significant canonical functions yielded between the anthropometric dimension (%BF-SKF, %BF-BIA, BMI, WC, and WHtR) and the cardiorespiratory fitness dimension (one-mile run and PACER). The first canonical function had a coefficient of *R*_*c*_ = .695, *P* < .001, and the second canonical function had a coefficient of *R*_*c*_ = .317, *P* < .001. The Rd for the first canonical function was Rd = .373, or 37.3% of shared variance; the Rd for the second significant canonical function was Rd = .0227, or 2.27% of shared variance. Because the first canonical function explained a significantly greater amount of shared variance between the two variates, it was the only canonical function justified for further analysis and interpretation.  Table 3 shows the raw and standardized coefficients, canonical loadings, and canonical crossloadings for each measure for the first canonical function. The first canonical correlation shows that WHtR had the highest standardized coefficient in the anthropometric dimension (.797) with %BF-SKF, %BF-BIA, and WHtR having the three strongest canonical loadings (.747, .817, and .603, resp.) and crossloadings (.519, .568, and .419, resp.). The one-mile run had the highest standardized coefficient (.734), highest canonical loading (.951), and highest cross loading (.661) in the cardiorespiratory fitness dimension. These results suggest that the anthropometric and cardiorespiratory fitness dimensions do indeed correlate strongly with one another, with %BF-SKF, %BF-BIA, and WHtR measures significantly associating with the anthropometric and cardiorespiratory fitness dimensions. 

### 3.3. Pearson and Partial Correlations

 Tables 4 and 5 display the Pearson correlation matrices for all anthropometric measures and cardiorespiratory fitness scores for girls and boys, respectively. All anthropometric measures significantly associated with each other in both genders yielding moderate to moderately high correlations among the parameters (*P* < .001). One-mile run times correlated significantly with PACER scores for both boys (*r* = −.655, *P* < .001) and girls (*r* = −.415, *P* < .001). These inverse correlations suggest that faster one-mile run times were associated with higher PACER scores (an increase in performance), and vice-versa. %BF-SKF, %BF-BIA, and WHtR significantly correlated with one-mile run times in both genders (*P* < .001) yielding similar magnitude “moderate” correlations. WC only correlated significantly with one-mile times in girls (*r* = .412, *P* < .001). Likewise, BMI only significantly correlated with one-mile run times in girls (*r* = .280, *P* < .05). The direct (positive) correlations suggest that higher anthropometric measures were associated with slower one-mile run times (a decrease in performance), and vice-versa. In general, the anthropometric measures correlated less strongly to PACER scores than the one-mile times. In boys, PACER scores only significantly correlated with %BF-SKF (*r* = −.440, *P* < .001) and with %BF-BIA (*r* = −.477, *P* < .001). In girls PACER scores correlated with %BF-SKF (*r* = −.315, *P* < .001), %BF-BIA (*r* = −.288, *P* < .05), WC (*r* = −.262, *P* < .05), and WHtR (*r* = −.305, *P* < .05). The inverse (negative) correlations suggest that higher anthropometric measurements were associated with lower PACER scores (a decrease in performance), and vice-versa. Using partial correlations to control for BMI and age (Table 6), similar correlations were found between the anthropometric measures and cardiorespiratory fitness test scores in both genders, with BMI and age seemingly having little confounding effect on the relationships.

## 4. Discussion

 The primary aim of this study was to examine the associations between anthropometric measurements and cardiorespiratory fitness test performance in middle-school students. Results from the canonical correlation analysis suggest that there was a moderate to strong relationship between the anthropometric dimension and the cardiorespiratory fitness dimension. %BF-SKF and %BF-BIA along with WHtR had the strongest associations with the anthropometric dimension and the cardiorespiratory fitness dimension in this sample. Pearson product moment correlations indicated that %BF-SKF and %BF-BIA had moderate associations with one-mile run and PACER scores in both genders. WHtR had moderate associations with one-mile run in both genders and a significant association with PACER scores in girls. WC had statistically significant associations with one-mile run and PACER in girls, and BMI only had a statistically significant association with the one-mile run in girls. After controlling for BMI and age in this sample, most of the aforementioned significant correlations held, with BMI and age seemingly having little confounding effect on the relationships between anthropometric measures and cardiorespiratory fitness test scores.

 In general, the differences among the grades and between the genders were consistent with previous research where cardiorespiratory fitness test scores improved at higher grade levels, boys showing lower mean body fat percentages than girls, and boys performing better on tests of cardiorespiratory fitness [27]. WC measurements were higher in boys compared to girls, despite having overall lower body fat percentages, but the differences between the genders were statistically nonsignificant. A higher mean WC in boys may be due to body fat deposits tending to accumulate in the abdominal region in males as opposed to the hips and buttocks as it tends to do in females during physical maturation [28, 29]. Paradoxically, however, WC and WHtR had the strongest associations with the cardiorespiratory fitness test scores in girls as opposed to boys, where body fat estimations had the highest associations with cardiorespiratory fitness. One possible explanation for this is the higher amount of lean body mass that tends to accumulate in boys during adolescence. A WC measurement does not distinguish between central adiposity and muscle (lean body mass); therefore, males with higher muscle mass may have an increased WC measurements not entirely due to fat accumulation but rather at least partially due muscular core development. Despite this possible limitation of WC, the correlations between WHtR were stronger than BMI across the cardiorespiratory fitness scores in both genders, displaying similar associations with the fitness scores as the associations between the body fat measurements and fitness. WHtR is useful because it takes into account an individual's height when estimating central adiposity. This is an important consideration because as children progress through development and into adulthood, bone structure changes make WC a less useful tool when comparing potential health risk between subjects. One student may be early in his or her development yet have a WC measurement similar to a peer who is taller, more physically developed, and in better physical condition. In this situation, the WC would be a less valid tool because of the height and physical development contrasts between subjects. One way to account for this is using age-gender reference tables; however, WHtR may provide a simpler index for interpretation purposes. The correction for height that WHtR takes into account would suggest that the taller individual would have less of a risk of cardiometabolic disease than the shorter individual with the same waist circumference based on previous research [30]. The results of this study support the evidence that WHtR may be a more useful indicator of cardiorespiratory fitness yielding stronger correlations with the cardiorespiratory fitness test scores than WC in both girls and boys. 

 The associations between cardiorespiratory fitness and health, specifically cardiovascular health, have been established in the literature, and body composition has also shown associations with health in both children and adults [31–33]. The canonical correlation analysis in this study supports that the anthropometric dimension, which comprises measures that estimate body composition, had moderate to strong correlations with the cardiorespiratory fitness dimension in the first canonical function. Within the anthropometric dimension, WHtR, %BF-SKF, and %BF-BIA had the highest standardized coefficients, canonical loadings, and crossloadings. Canonical correlations maximize the associations between two dimensions or variates, which are a weighted linear combination of variables. Results from this analysis show that WHtR, %BF-SKF, and %BF-BIA had the strongest associations with both the anthropometric dimension and the fitness dimension. WC and BMI had weaker standardized coefficients and loadings in the model compared to the other aforementioned measures. This may suggest that BMI and WC may provide a less useful indicator of overall body composition than the other measures in the dimension. As stated previously, BMI limitations include that it does not account for lean body mass in an individual nor does it specify fat distribution. WC's major limitation is that it does not take into consideration height or bone structure, which makes comparisons between individuals difficult when identifying health risk. %BF-SKF, %BF-BIA, and WHtR take these aforementioned limitations into account, which is reflected by their stronger associations with cardiorespiratory fitness test performance in this sample.

 This study manifests some practical implications that must be considered for professionals who assess health and cardiorespiratory fitness in youth. Due to the stronger associations that body fat estimated from %BF-SKF, %BF-BIA, and WHtR had with the cardiorespiratory fitness scores compared to BMI, these measures may be preferable to BMI when attempting to identify children with less than adequate physical fitness. Administering the two-site SKF assessment to estimate body fat, although it is a better indicator of overall body fatness than BMI, can be cumbersome especially if used in a physical education setting for a large group of children. SKF may also make certain children feel uncomfortable during the assessment due to skin pinching. BIA certainly provides an alternative to the two-site skinfold for the estimation of body fat. However, research is conflicting on the agreement between these two methods in different populations of youth [34]. BIA, although useful for describing body composition in groups, its %BF estimates have large errors in individuals being influenced by factors such as nutritional and hydrational status [27]. WHtR offers an alternative to both these methods. Although it is not as direct of a method to approximate overall body fatness, WHtR estimates an individual's central adiposity relative to their height. Central adiposity has been associated with higher risk of chronic disease than overall body fatness in children and adults. The results from this study suggest that WHtR moderately associates with cardiorespiratory fitness in middle school-students. WHtR is also easy to administer, as all one needs is a standard tape measure and the height of the individual. And unlike WC, WHtR may not need age-gender reference tables for interpretation once a standard has been established for a population. Using this index can be used in clinical settings in attempting to identify children who are at higher risk for developing chronic disease. In physical education class, it could be used as an alternative to BMI throughout the school year for body composition assessment, or to assess the effectiveness of various curricula or interventions aimed at improving the health status of a class. Only 37% of states require some form of assessment in physical education, and of those that do, only 74% require assessment of physical fitness [35]. For those significant number of schools in the US that do not have some form of physical education body composition assessment, the WHtR index provides a valid, efficient, and easy-to-use measure for school nurses and health specialists to assess health of their respective student population outside of physical education.

 There are limitations of this study that affect the generalizability of the results. The sample consisted of students in the 6th, 7th, and 8th grades from institutions where the racial distribution was heavily Caucasian (sample was approximately 85% Non-Hispanic Caucasian). Future research needs to examine the anthropometric associations with cardiorespiratory fitness test performance in all age groups and from a more ethnically diverse sample of children. Also, approximately 24% of the sample was classified as overweight/obese by FITNESSGRAM's body composition standards [36]. Future research may need a better representation of overweight/obese children when examining the associations in this study. Finally, WHtR currently has no established standards for US children in any age group, unlike BMI and body fat percentage. Future research may consider the findings of this investigation to inspire a larger scale study to set criterion referenced standards for WHtR to provide meaning to the interpretation of this index. 

 In conclusion, body fat estimated from the two-site SKF and the hand-held Omron BIA device, as well as WHtR, had moderate associations with cardiorespiratory fitness in middle-school students. BMI and WC had weaker associations with cardiorespiratory fitness test performance. The results of this study suggest that estimating body fat from either SKF or BIA, or accounting for height when measuring waist circumference (WHtR), may offer more valid alternatives to BMI and WC when trying to identify children that have less than adequate cardiorespiratory fitness, a strong indicator of health status. Future research needs to further explore these associations on different populations of children to establish screening measures that most accurately identify children at risk for chronic disease. 

## Figures and Tables

**Table 1:** Anthropometric measures and cardiorespiratory fitness scores per grade and gender group (means ± S.D.).

	Grade 6	Grade 7	Grade 8	Girls	Boys
	(*n* = 34)	(*n* = 52)	(*n* = 48)	(*n* = 69)	(*n* = 65)
Anthropometric measures					
Height (m)	1.52 ± .066	1.63 ± .104^+^	1.69 ± .085^+^	1.59 ± .08	1.64 ± .12*
Weight (kg)	40.6 ± 7.26	49.7 ± 10.0	60.9 ± 12.8^+^	47.78 ± 9.92	52.69 ± 13.60*
BMI (kg/m^2^)^1^	17.4 ± 2.95	18.4 ± 2.59	21.0 ± 3.07^+^	18.69 ± 2.87	19.18 ± 3.21
% Body fat (SKF)^2^	20.7 ± 6.95	20.3 ± 6.64	21.3 ± 9.05	23.35 ± 5.99	19.30 ± 7.85*
% Body fat (BIA)^3^	21.7 ± 6.52	19.2 ± 5.89	19.2 ± 19.2	22.08 ± 5.09	18.38 ± 6.84*
WC (cm)^4^	63.5 ± 6.13	66.8 ± 7.77	72.8 ± 8.27^+^	65.89 ± 7.91	68.46 ± 8.10
WHtR^5^	.416 ± .042	.409 ± .045	.429 ± .039	.413 ± .045	.415 ± .041
Cardiorespiratory fitness scores					
One mile time (s)	561.0 ± 145.7	522.1 ± 142.8	426.7 ± 91.34^+^	557.2 ± 139.0	450.4 ± 100.6*
PACER (laps)	50.0 ± 19.3	49.2 ± 21.4	65.5 ± 26.1	44.79 ± 18.60	60.4 ± 23.04*

^1^BMI stands for Body Mass Index.

^2^SKF stands for % body fat estimation from the two-site skinfold method and Slaughter formula.

^3^BIA stands for % body fat estimation from the Omron BIA device.

^4^WC stands for waist circumference.

^5^WHtR stands for waist-to-height ratio.

^+^Grade effect, *P* < .05.

*Gender effect, *P* < .05.

**Table 2:** Canonical correlations.

Canonical correlation	Wilks' lambda	Degrees of freedom 1	Degrees of freedom 2	*F* statistic	Significance
.695	.4649	10	254	11.85	*P* < .001
.317	.8993	4	128	3.580	*P* < .001

**Table 3:** Canonical coefficients and loadings for first canonical function.

	First canonical function				
	Raw canonical coefficient	Significance	Standardized canonical coefficient	Canonical loading	Canonical cross-loading
Anthropometric dimension					
BMI^1^	−.177	*P* < .001	−.540	.224	.155
BF-SKF^2^	.063	*P* < .001	.454	.747	.519
BF-BIA^3^	.079	*P* < .001	.497	.817	.568
WC^4^	−.127	*P* = .05	−.404	.258	.179
WHtR^5^	18.52	*P* < .001	.797	.603	.419
Cardiorespiratory fitness dimension					
One-mile time	−.017	*P* < .001	.734	.951	.661
PACER	.005	*P* < .001	−.374	−.800	−.556

^1^BMI stands for Body Mass Index.

^2^SKF stands for % body fat estimation from the two-site skinfold method and Slaughter formula.

^3^BIA stands for % body fat estimation from the Omron BIA device.

^4^WC stands for waist circumference.

^5^WHtR stands for waist-to-height ratio

**Table 4:** Correlation matrix showing anthropometric measures and cardiorespiratory fitness scores for girls.

	BMI	%BF (SKF)	%BF (BIA)	WC	WHtR	One-mile time	PACER score
BMI^1^	1						
%BF (SKF)^2^	.651**	1					
%BF (BIA)^3^	.604**	.583**	1				
WC^4^	.755**	.559**	.475**	1			
WHtR^5^	.708**	.530**	.573**	.889**	1		
One-mile time	.280*	.412**	.400**	.412**	.583**	**1**	
PACER	−.194	−.315**	−.288*	−.262*	−.305*	−.415**	**1**

^1^BMI stands for Body Mass Index.

^2^SKF stands for %body fat estimation from the two-site skinfold method and Slaughter formula.

^3^BIA stands for %body fat estimation from the Omron BIA device.

^4^WC stands for waist circumference.

^5^WHtR stands for waist-to-height ratio.

*P < .05.

**P < .001.

**Table 5:** Correlation matrix showing anthropometric measures and cardiorespiratory fitness scores for boys.

	BMI	%BF (SKF)	%BF (BIA)	WC	WHtR	One-mile time	PACER score
BMI^1^	1						
%BF (SKF)^2^	.612**	1					
%BF (BIA)^3^	.561**	.762**	1				
WC^4^	.820**	.522**	.523**	1			
WHtR^5^	.707**	.625**	.632**	.803**	1		
One-mile time	.199	.449**	.606**	.203	.431**	**1**	
PACER	−.004	−.440**	−.477**	−.032	−.153	−.655**	1

^1^BMI stands for Body Mass Index.

^2^SKF stands for %body fat estimation from the two-site skinfold method and Slaughter formula.

^3^BIA stands for %body fat estimation from the Omron BIA device.

^4^WC stands for waist circumference.

^5^WHtR stands for waist-to-height ratio.

*P < .05.

**P < .001.

**Table 6:** Partial correlations controlling for BMI and age.

	One-mile time		PACER	
	Boys	Girls	Boys	Girls
% BF (SKF)^1^	.393**	.316*	−.542**	−.262*
% BF (BIA)^2^	.549**	.164	−.539**	−.196
WC^3^	.146	.328*	.034	−.170
WHtR^4^	.412**	.470**	−.202	−.241*

^1^SKF stands for % body fat estimation from the two-site skinfold method and Slaughter formula.

^2^BIA stands for % body fat estimation from the Omron BIA device.

^3^WC stands for waist circumference.

^4^WHtR stands for waist-to-height ratio.

*P < .05.

**P < .001.
